# Perspectives on CMIP5 model performance in the Nile River headwaters regions

**DOI:** 10.1002/joc.4284

**Published:** 2015-02-13

**Authors:** Partha S. Bhattacharjee, Benjamin F. Zaitchik

**Affiliations:** ^1^ I.M. Systems Group, Inc.NOAA/NCEP Environmental Modeling CenterCollege ParkMDUSA; ^2^ Department of Earth and Planetary SciencesJohns Hopkins UniversityBaltimoreMDUSA

**Keywords:** CMIP5, GCM, climate change, Nile River, water resources

## Abstract

Ranking the performance of global climate models (GCMs) is a notoriously difficult exercise. Multi‐model comparison studies nearly always show that each model has strengths and weaknesses relative to others, and for many purposes the multi‐model ensemble mean delivers better estimates than any individual model. Nevertheless, in regions like East Africa, where there is little consensus between models on the magnitude or sign of 21st century precipitation change, the multi‐model ensemble mean approach to climate projection provides little value for adaptation planning. Here, we consider several possible frameworks for model evaluation and ranking, and assess the differences in performance of a subset of models participating in the 5th Coupled Model Intercomparison Project (CMIP5) according to each framework. Our test case is precipitation in the Nile River headwaters regions. We find that there is little consistency in the relative performance of models across frameworks based on amount and seasonality of precipitation, interannual precipitation variability, precipitation teleconnections, and continental scale climate patterns. These analyses offer some guidance on which GCMs are most likely to provide meaningful results for specific applications, but they caution that any effort to select ‘best performing’ GCMs for the Nile River basin must carefully consider the purposes for which GCMs are being selected.

## 1 Introduction

Global climate models (GCMs) are regularly applied to study past, present, and future climate in Africa (Braconnot *et al.*, 2012; Biasutti, 2013; Otieno and Anyah, 2013; Rowell, 2013; Tierney *et al.*, 2013; Müller *et al.*, 2014). These studies have characterized dominant dynamical processes, provided insights on the geologic and recent historic records, and framed our understanding of how future climate change might impact the continent. At the same time, multi‐model GCM studies of African climate as a whole and of selected sub‐regions have consistently shown that GCMs differ dramatically in their representation of precipitation climatology and variability, even within the period of modern observations (IPCC, 2013; Otieno and Anyah, 2013; Rowell, 2013; Jury, 2015).

This lack of consensus projects onto simulations of future climate: while models generally agree on the direction of precipitation change in parts of Africa directly affected by Hadley Cell strengthening [e.g. North Africa, some portions of Equatorial Africa (IPCC, 2013)], there is wide model disagreement in climatically complex regions such as the Greater Horn of Africa (GHA), parts of southern Africa, and the Sahel (Williams and Funk, 2011; Biasutti, 2013; Otieno and Anyah, 2013). Collectively, these regions of large uncertainty comprise most of the continent. Africa is not unique in this regard, as resource managers and policy makers struggle with the problem of uncertain precipitation projections in many regions (Kundzewicz and Stakhiv, 2010). But in tropical Africa the disagreement between models is particularly large, and observations available for model parameterization and evaluation are relatively limited (Brands *et al.*, 2013). This is troubling, as the regions of greatest model disagreement – including the Sahel and the GHA – are particularly vulnerable to climate variability and change (Müller *et al.*, 2014).

In this context, there is considerable interest in explaining the lack of GCM consensus for present and future African climate and, if possible, narrowing the multi‐model ensemble spread by selecting only the most reliable models as the basis for climate projections. A number of recent studies have sought to evaluate the relative performance of GCMs participating in the 3rd and 5th phases of the Coupled Model Intercomparison Project (CMIP3 and CMIP5; Taylor *et al.*, 2012) for selected regions of Africa. Some of these studies have focused on process representation (Williams and Funk, 2011; Dirmeyer *et al.*, 2013; Roehrig *et al.*, 2013) while others have implicitly or explicitly ranked GCMs based on their ability to replicate statistics of precipitation (Otieno and Anyah, 2013; Jury, 2015), teleconnections (Rowell, 2013; Martin *et al.*, 2014), or large scale atmospheric fields (Brands *et al.*, 2013) in simulations of 20th century climate.

Any attempt to rank GCM performance in a region of interest must be approached with caution. From a practical standpoint, it is possible that a model that performs well for regional precipitation performs relatively poorly on temperature variability, or that a model that captures the dynamics of variability in one part of a region of interest does not capture the way in which these dynamics influence the rest of the region. Perhaps even more importantly, our observations of climate – even if they were perfect – represent only one realization of a semi‐chaotic system. For example, it is possible that a particular region of interest has been affected by a multidecadal pattern of variability in the second half of the 20th century. There is no reason to believe that a GCM – even a perfect GCM – would exhibit that same mode of long‐term variability at the same time: the CMIP5 historical simulations are not initialized from any historically accurate set of initial conditions, so the occurrence of climate oscillations is random relative to the actual historical record. We can assume that this will average out for relatively short period oscillations, but variability on the scale of decades to centuries might cause a particular model simulation to be ‘biassed’ relative to historical observations simply because it is in a different phase of variability throughout the observational record. Large ensemble GCM simulations offer an opportunity to examine the influence of internal climate variability on these time scales, but the publicly available CMIP5 archive does not contain adequate model output to perform a robust evaluation. These issues, combined with the fact that GCM outputs are used for different purposes by different users, pose significant challenges to any effort to distinguish high performing models from low performing models (Giorgi and Mearns, 2002; Tebaldi and Knutti, 2007; Stephenson *et al.*, 2012).

Nevertheless, the impetus to establish some kind of ranking and model selection for climatically sensitive regions of Africa is clear. It is difficult to accept a ‘one model one vote’ multi‐model ensemble average approach to climate projection when model performance is so variable and the potential impacts of climate change are so severe. Moreover, it is known that precipitation projections of many GCM projections are at odds with observations in recent decades in vulnerable regions such as the Horn of Africa (Williams and Funk, 2011). In this context, if one is to use GCMs for future climate projection at all then it is clearly desirable to identify which GCMs reliably capture which features of climate before making any conclusions about likely future climate change. Given the challenges listed above, however, it is important to understand that any ranking of GCMs is really a ranking of a particular simulation of the GCM, which is a product of both the model and internal variability that is sensitive to initialization procedures.

Bearing these limitations in mind, we consider three conceptual frameworks that might be applied to select best‐performing GCMs for purposes of climate projection in Africa, using precipitation projections for the Nile River headwaters regions as a case study: the Upper Blue Nile (UBN) Highlands of Ethiopia for the Blue Nile and the Equatorial Lakes (EQL) region for the White Nile. We focus on these two regions because they are collectively responsible for the majority of rainfall in the Nile River basin and because they are located in different climate zones with distinctly different patterns of variability. In analyzing GCM projections for the Nile River basin we follow on several previous studies (Kim and Kaluarachchi, 2009; Beyene *et al.*, 2010; Taye *et al.*, 2010). But here we consider the Nile as a test case for how different frameworks for GCM analysis influence assessment of historical model performance and, by association, perceived reliability for projecting future climate change.

The frameworks we consider are as follows. First, we examine the performance of a selection of CMIP5 models on standard statistical metrics of precipitation – mean and variability in the major rainfall seasons and seasonality. This is similar to previous studies that have assessed model performance for Ethiopia (Jury, 2015) and the GHA (Otieno and Anyah, 2013). In the absence of a large ensemble of simulations for each GCM, this approach cannot definitively distinguish between model physics and multidecadal or century‐scale internal variability. Results must be interpreted as an evaluation of a particular model realization rather than of a modelling system in general.

Second, we evaluate the representation of known teleconnections affecting East Africa in GCMs relative to observations. This approach is often adopted in model evaluation because GCMs capture large scale climate phenomena more reliably than local processes. If a GCM is to be used to project future climate conditions, it can be argued that it is more important that it captures regional variability associated with major climate features than that it captures the exact amount or seasonal timing of precipitation at a location of interest. GCM precipitation is a diagnostic field that can be bias corrected, whereas an inability to simulate the connection between Indian Ocean SST and variability in East African precipitation, for example, suggests that a model fails to capture basic climate dynamics that are relevant to observed variability of 20th century climate and might change as climate evolves over the 21st century. In evaluating CMIP5 representation of teleconnections affecting Africa we follow Rowell (2013), who performed a comprehensive teleconnection analysis for several regions across the continent, and Martin *et al.* (2014) who examined teleconnections affecting the Sahel. This approach to model evaluation can also be affected by long‐term internal variability, such as the interaction between multidecadal oscillations with higher frequency modes like ENSO.

Finally, we briefly consider GCM representation of African precipitation variability at continental scale. Following Giannini *et al.* (2008), we examine the primary components of Africa‐wide precipitation variability in a subset of GCMs. As each of these components can be correlated with global SST patterns, we can investigate whether a GCM that performs well in the Nile basin also captures global drivers of African precipitation in general. This is relevant in a nonstationary climate system, as models that offer a realistic representation of the relative strength of climate phenomena across the continent may be viewed as more reliable when simulating shifts in these dynamics over time.

We do not claim that any one of these frameworks is absolutely better than the others, nor do we consider them to be a comprehensive review of methods of GCM evaluation. The objective of the paper is simply to present multiple frameworks for model selection in a single, consistent study and to demonstrate that different model selection frameworks can lead to very different choice of GCMs. For this reason, the decision of how to select GCM realizations for any given application should be made in the context of study design and the objectives of the model user.

## 2 Data and methodology

We draw precipitation data for the UBN and EQL regions from the Climate Research Unit (CRU) Time Series 3.2 (TS3.2) monthly gridded precipitation dataset (Harris *et al.*, 2013). The period of analysis (1950–1995) was selected on the basis of number of meteorological stations contributing to the gridded CRU data set: as noted in previous studies (Rowell, 2013; Badr *et al.*, 2014), the number of stations reporting to CRU in Africa drops precipitously after the mid‐1990s. We select a period that has a large number of reporting stations across Africa and is long enough to characterize major patterns of interannual to interdecadal variability. The Met Office Hadley Centre Sea Ice and Sea Surface Temperature monthly‐mean dataset (HadISST) (Rayner *et al.*, 2003) is used for observational SST data, and NCEP/NCAR Reanalysis Project data (Kalnay *et al.*, 1996) were used for atmospheric fields.

For climate models, we use output from ten different coupled Atmosphere–ocean Global Climate Models (AOGCMs) participating in CMIP5 (source: http://pcmdi9.llnl.gov/). For each model we use the ‘historical’ simulations, which are forced with observed aerosol and greenhouse gas concentrations from 1850–2005, and 21st century simulations generated using high emissions Representative Concentration Pathway (RCP8.5; Moss *et al.*, 2010). Monthly precipitation, pressure level zonal wind (U‐wind) and SST output fields from both of these experiments are used and only the first member of the ensemble simulations is utilized in order to provide consistent statistics across models. The period of analysis is 1950–1995 for historical simulations. Models used in this study are BCC‐CSM1‐1 (BCC, China), CCSM4 (NCAR, USA), CESM1 (NCAR, USA), CSIRO‐Mk3.6 (CSIRO, Australia), CanESM2 (CCCMA, Canada), GFDL‐ESM2M (NOAA GFDL, USA), GISS‐E2‐R (NASA GISS, USA), HadGEM2‐ES (Hadley Center, UK), IPSL‐CM5A‐LR (IPSL, France), and MIROC5 (JAMSTEC, Japan). These ten models represent a reasonable spread across model genealogy in the CMIP5 ensemble (Knutti *et al.*, 2013). We note that the relatively coarse resolution of GCMs means that the models generally fail to capture observed topographically driven variability in the study region.

**Figure 1:**
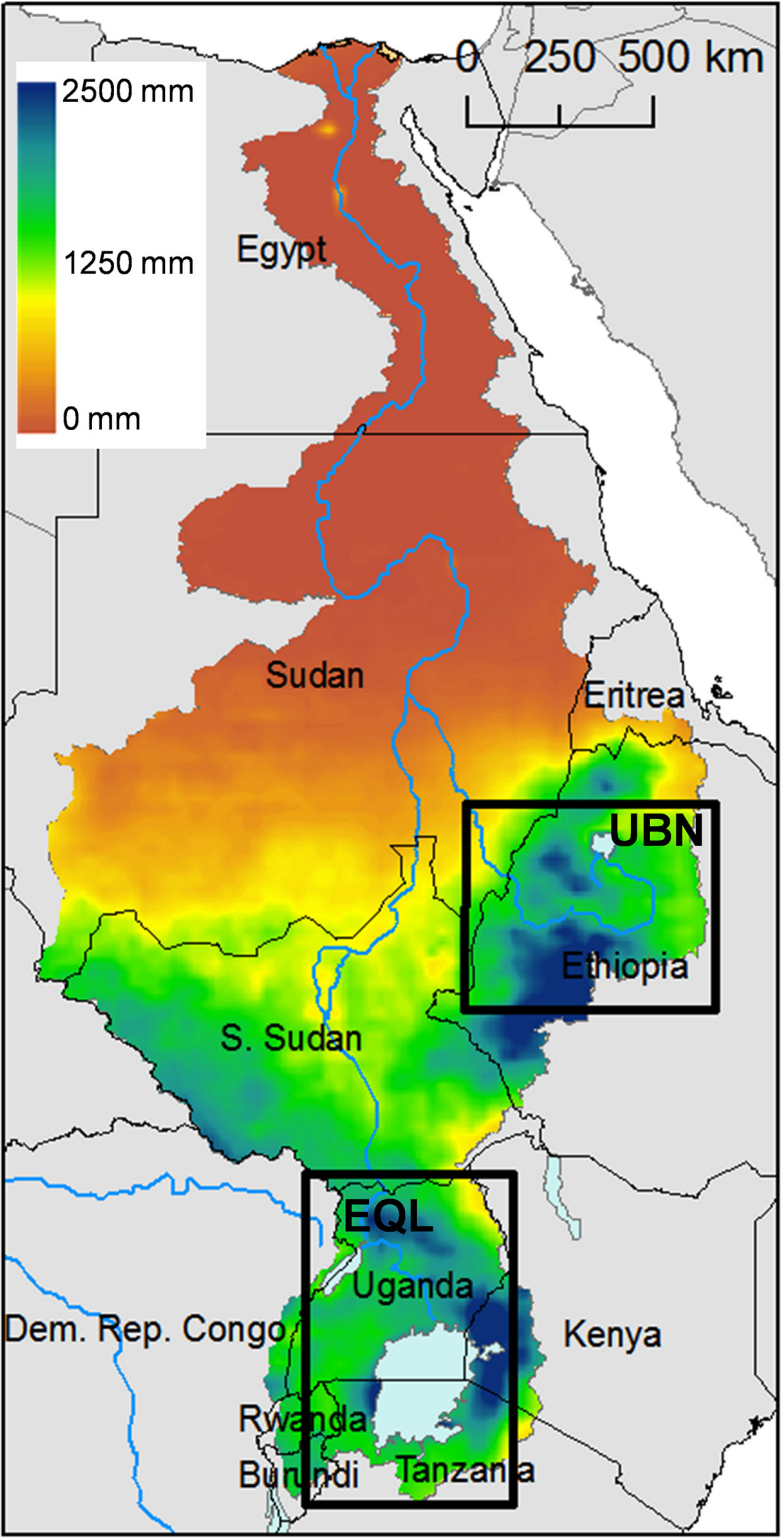
The Nile River basin, with the Equatorial Lake (EQL) and Upper Blue Nile (UBN) headwaters regions shown in boxes.

The Nile River headwaters regions are defined by approximate boxes, consistent with the resolution of CMIP5 data (Figure 1). For the UBN (8°–12°N, 34°–40°E) we aggregate data across the June–September (JJAS) rainy season and for EQL (5°S–4°N, 30°–35°E) we consider both the March–May ‘long rains’ (MAM) and the October–December ‘short rains’ (OND). We also analyze monthly precipitation throughout the year to examine seasonality in GCMs relative to observations. We choose these regions because they are critical to Nile River trans‐national fresh water resources and are climatically distinct from one another. We recognize, however, that there is considerable intraregional variability within both the UBN and EQL. In addition, there is substantial intraseasonal variability in the strength of large‐scale teleconnections for each region (Berhane and Zaitchik, 2014; Berhane *et al.*, 2014). These heterogeneities compromise the statistical strength of our analysis, but we accept this simplification because our purpose is to assess general implications of model evaluation framework for the Nile basin rather than to optimize for prediction or dynamically based explanation.

For teleconnections analysis, we examine linear correlations between seasonal precipitation and four indices that are commonly associated with precipitation variability in the Nile basin (Camberlin, 1997, 2009; Anyah and Semazzi, 2006; Block and Rajagopalan, 2007; Segele et al., 2009; Kundzewicz and Stakhiv, 2010; Diro et al., 2011; Berhane et al., 2014): (1) an ENSO index computed from the 3‐month running means of SST anomalies between 10°S–10°N and 120°E–80°W, (2) Global SST anomaly (GSST), as an indicator of global‐scale climate variability and change (in this study SST between 60°N–60°S are considered), (3) a dynamically based Indian Summer Monsoon Index (ISM; http://apdrc.soest.hawaii.edu/projects/monsoon) calculated as the difference of zonal wind at 850 mbar between region 1 (5°–15°N and 40°–80°E) and region 2 (20°–30°N and 70°–90°E) for the JJAS season (Wang and Fan, 1999), and (4) an Indian Ocean Dipole (IOD) index calculated as the difference between the tropical western Indian Ocean (50°–70°E, 10°S–10°N) and the tropical south‐eastern Indian Ocean (90°–110°E, 10°S–0°N) (Saji et al., 1999). All anomalies are calculated from the 1950–1995 climatology.

We analyze continental scale patterns of variability using an approach similar to Giannini et al. (2008): we calculate the first three principal components (PC) of interannual precipitation variability for all of Africa – defined as all land area between 40°S–40°N and 20°W–60°E, and for a year that runs from July to June – map spatial patterns of PC correlations with gridded precipitation, and then correlate the time series of the PCs with the global SST field. For continental analysis we use annual precipitation and SST and use the same historical period of analysis as in all other analyses (1950–1995).

## 3 Results and discussion

### 3.1 Statistics of precipitation

First, we consider GCM representation of the basic statistics of precipitation in the UBN and EQL: mean rainy season precipitation, seasonality, and interannual variability. Related statistics have been used for GCM selection in the Ethiopian Highlands (Jury, 2015), the GHA (Otieno and Anyah, 2013), and for presentation of GCM projections worldwide (IPCC, 2013). Considering bias, we note that almost all models included in this study exhibit a wet bias for the EQL short rains (OND), while biases vary widely between models for the EQL long rains (MAM) – ranging from a 62% dry bias to a 55% wet bias – and the UBN – ranging from a 55% dry bias to a 99% wet bias. In all three seasons GISS is the driest model and MIROC5 is the wettest. When we consider representation of interannual variability, we see that the majority of models overestimate variability (as a percent of total precipitation) in the EQL region for both seasons and underestimate variability in the UBN.

If one were to select GCMs based on their ability to replicate statistics of mean precipitation and interannual variability, then, the results shown in Table 1 favour using BCC and CESM1 for the EQL in MAM, GISS for the EQL in OND, and CCSM4 for the UBN. It is noteworthy that no single model excels in both water catchment areas or in both rainy seasons within the EQL.

**Table 1:** 1950–1995 mean precipitation (mm day^–1^) and interannual variability (expressed as variance as a percent of mean) for CRU and CMIP5 models

	EQL (MAM)		EQL (OND)		EQL (Clim)	UBN (JJAS)		UBN (Clim)
	Mean	Variability (%)	Mean	Variability (%)	Seasonality	Mean	Variability (%)	Seasonality
CRU	4.51	42	3.56	67	–	7.42	80	–
BCC‐CSM1‐1	**4.73**	**58**	7.46	**39**	0.27	**4.79**	25	0.70
CCSM4	**3.26**	96	7.68	129	0.56	**6.01**	**60**	0.69
CESM1(CAM5)	**4.62**	**55**	7.22	124	0.53	**6.46**	**37**	**0.97** *
CSIRO‐Mk3.6	**6.15**	188	5.96	304	**0.84**	**7.27**	**43**	**0.85** *
CanESM2	**4.17**	105	6.4	**113**	0.65	**8.87**	15	**0.95** *
GFDL‐ESM2M	**2.51**	109	5.77	207	0.52	**7.58**	19	0.68
GISS‐E2‐R	1.71	**51**	**2.82**	**44**	0.43	3.34	**53**	**0.85**
HadGEM2‐ES	**3.19**	**41**	5.76	**106**	0.28	**6.87**	18	**0.97** *
IPSL‐CM5A‐LR	**3.87**	120	**5.17**	137	0.42	**4.73**	**66**	**0.97** *
MIROC5	7.01	**45**	8.28	**32**	0.69	14.8	**88**	**0.97** *
Multi‐model averages	4.12	87	6.25	124	0.596	7.07	42.4	0.923

Values for which model and CRU values agree within 25% are indicated in bold italics. Agreement within 50% is in bold. Disagreement >100% is in italics. For ‘seasonality’, correlations were calculated between the climatological monthly precipitation values for each CMIP5 model those of CRU. Correlation coefficients >0.9 are in bold italics, >0.8 are bold, <0.5 are italicized, and

* indicates statistical significance at a = 0.05, accounting for temporal autocorrelation. Both observational and model data are linearly interpolated to a common 1° × 1° grid box.

**Figure 2:**
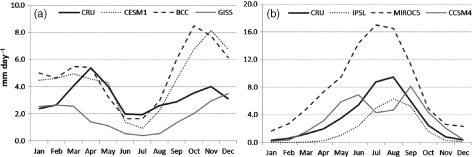
Climatological monthly precipitation, 1950–1995, for CRU and selected CMIP5 model historical simulations in (a) EQL and (b) UBN.

**Figure 3:**
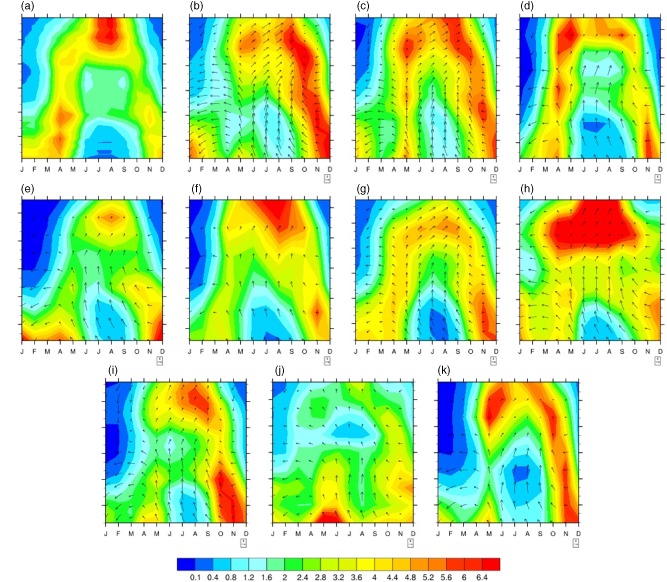
Zonally averaged monthly precipitation (mm day^–1^) and wind fields (both zonal and meridional components in m s^–1^ at 925 mb) among models and CRU data. (a) CRU, (b) CCSM4, (c) CESM, (d) CSIRO, (e) IPSL, (f) CanESM2, (g) BCC, (h) MIRCC5, (i) HadGem2, (j) GISS, and (k) GFDL. Climatological monthly means between 1950 and 1995 are used for all the fields between 6°S and 12°N. Averages between 30°E and 45°E longitudes are calculated for zonal means. Referenced wind vector magnitude is 4 m s^–1^ in the figure. All data are in their original resolution.

Moreover, additional concerns arise when one includes a model's ability to capture the seasonal cycle as an additional criterion of evaluation. Here we evaluate a model's seasonality as the correlation of climatological monthly precipitation – i.e. the annual cycle – with CRU (Table 1). In this region, a model's representation of precipitation seasonality is primarily a function of its simulation of the migration of the Inter‐tropical convergence zone (ITCZ). As other authors have noted, the ITCZ has been diagnosed in terms of surface convergence, cloud‐top height, or surface pressure and precipitation, and these diagnostics do not always align (Nicholson, 2013). Here we use the location of the tropical rain‐belt as a proxy for the ITCZ (Zhang et al., 2006; Nicholson, 2009), because our focus is on model representation of precipitation. In the EQL, BCC, GISS, and CESM all fail to replicate the annual cycle (to varying degrees) because they do not realistically simulate the dry period in January and February (Figure 2(a)). As shown in Figure 3, this problem arises from the fact that the tropical rain belt in these models simply never moves far enough south to allow for a dry winter period. In Figure 3, zonally averaged rainfall and low level winds (at 925 mb) between 6°S–12°N is plotted using 1950–1995 climatology.

The importance of this model failure depends on the application. It is, clearly, a problem for studies that look at annual total precipitation, and the fact that the models are not representing ITCZ migration accurately in itself presents a concern when applying models to project future climate – since the ITCZ is such a dominant dynamical phenomenon in the tropics, and since it interacts with synoptic and meso‐scale phenomena relevant to precipitation in East Africa (Nicholson, 1996), a model that does not place the ITCZ in the proper location during the rainy season may be unreliable in simulating changes in precipitation dynamics over time. But for applications focused exclusively on rainy season processes – e.g. crop viability or flood risk – it is possible that errors in the off‐season can be accepted provided that rainy season dynamics are represented in realistic fashion.

In the UBN, CCSM4 produces a bimodal precipitation pattern rather than a unimodal peak (Figure 2(b)). In this case the problem is, again, the migration of the ITCZ: the CCSM4 rain belt pushes too far north in boreal summer, such that UBN precipitation peaks in June and September (as the simulated ITCZ migrates through the UBN in each direction) rather than in July and August, as is the case in observations. So while the model does well on seasonally averaged statistics, it does not provide realistic representation of intraseasonal variability. On one hand this might be viewed as a second order concern for an application concerned with seasonal totals (e.g. water resource analysis). However, relationships between large scale atmospheric processes and UBN precipitation evolve over the course of the rainy season, with Atlantic Ocean influences appearing in June and July and Pacific and Indian Ocean influences dominating in August and September (Berhane *et al.*, 2014). As these large scale features transform under climate change, a model that places the precipitation maximum in the wrong month within the rainy season might not provide a reliable estimate of how these transformations will influence precipitation in the UBN. Models such as IPSL and MIROC5, which have a dry and wet bias, respectively, but capture both variability and seasonality, might hold advantages in this regard.

### 3.2 Association with large‐scale drivers

While descriptive statistics of precipitation provide one view on GCM performance, they are not necessarily the most relevant consideration when selecting a model for impacts analysis or for dynamically based explanation. Standard bias correction and variance scaling methods exist and are often applied when using GCM precipitation fields for impacts studies (Wood *et al.*, 2004; Luo and Wood, 2008), and because precipitation is a diagnostic model output it is entirely possible for a model that captures prevailing dynamics correctly to have a precipitation bias due to parameterizations. Even seasonality errors can be accounted for through more advanced bias correction algorithms or by selective use of model output.

Instead, it can be argued that a better test of a model's performance is its ability to simulate the influence that large scale drivers of climate have on the region of interest. Doing so suggests that the model provides a meaningful representation of the influences that dominant climate dynamics have on a region, such that the model might be able to capture the ways in which changes at large scale will influence the region in the future. For this reason, we next consider teleconnections between UBN and EQL precipitation and ENSO, IOD, ISM, and GSST as simulated by the GCMs. Table 2 presents linear correlation coefficients between seasonal precipitation and each of these large scale climate indices for the period 1950–1995.

**Table 2:** Teleconnections between four large‐scale climate indices and precipitation in both EQL rainy seasons and the UBN rainy season

	ENSO			GLOBAL SST			ISM			IOD		
	EQL	EQL	UBN	EQL	EQL	UBN	EQL	EQL	UBN	EQL	EQL	UBN
	(MAM)	(OND)	(JJAS)	(MAM)	(OND)	(JJAS)	(MAM)	(OND)	(JJAS)	(MAM)	(OND)	(JJAS)
**CRU**	0.06	0.21	**−0.34**	0.04	0.09	**−0.32**	−0.01	−0.04	0.11	0.16	**0.46**	0.08
BCC	***−0.32***	−0.12	*0.24*	0.04	−0.01	*0.09*	0.26	0.04	***−0.31***	*−0.21*	0.21	0.09
CCSM4	−0.03	0.28	−0.07	0.11	0.22	−0.27	0.13	−0.05	***−0.3***	0.2	**0.41**	***−0.32***
CESM	0.11	−0.09	*0.01*	0.22	−0.15	−0.07	−0.2	**−0.36**	***−0.45***	−0.05	*−0.08*	0.06
CSIRO	−0.16	−0.11	*0.01*	−0.03	***−0.35***	−0.15	0.11	***0.36***	***0.47***	−0.15	*−0.16*	−0.1
CanESM	***−0.49***	0.21	−0.13	−0.16	0.01	*0.16*	0.12	−0.06	**0.31**	*−0.21*	0.16	−0.22
GFDL	***−0.32***	0.1	−0.09	−0.07	−0.07	−0.01	0.25	**−0.3**	−0.01	***−0.39***	**0.33**	−0.24
GISS	−0.03	−0.09	*0.24*	−0.06	0.09	0.01	0.07	−0.17	***0.47***	−0.06	*0.13*	*−0.28*
HadGEM	0.16	0.25	**−0.43**	0.11	−0.01	**−0.37**	−0.03	−0.23	***0.52***	0.23	**0.5**	−0.03
IPSL	*−0.28*	0.09	**−0.33**	−0.15	0.15	**−0.29**	0.11	0.01	0.24	*−0.25*	*−0.07*	−0.23
MIROC5	−0.04	−0.08	−0.18	−0.05	−0.07	−0.22	0.14	−0.14	0.23	0.18	*0.04*	0.06
Multi‐model averages	−0.05	0.25	0.03	0.29	0.05	−0.11	0.27	−0.07	0.34	0.11	0.17	−0.14

Strength of teleconnection is quantified as the linear correlation coefficient calculated for interannual variability over the period 1950–1995. Values in bold are different from zero at the 95% significance level. Values in italics are model results that differ from CRU at a significance level of 90% according to a two‐tailed Student *t*‐test on Fisher's *z*‐transformed values. All model data (both atmospheric and ocean parts) are linearly interpolated to a common 1° × 1° grid box.

Over this period, CRU precipitation estimates show the expected relationships with large scale processes. In UBN, there is a significant negative association between our ENSO index (in which positive values indicate El Nino conditions) and precipitation. This association has been noted in numerous studies (Tadesse, 1994; Camberlin, 1997; Conway, 2000; Gissila *et al.*, 2004; Segele and Lamb, 2005; Block and Rajagopalan, 2007; Segele *et al.*, 2009), with proposed mechanisms that include ENSO influence on the strength of southeasterly flow into East Africa from the Indian Ocean, ENSO modification of the African Easterly Jet and North‐African‐Asian Jet, and ENSO connections to the intensity of westerly winds that enter Africa from the tropical Atlantic Ocean. CRU also shows significant negative associations between UBN precipitation and global SST, which is consistent with patterns of association across the tropical ocean in previous studies (Berhane *et al.*, 2014). The statistical relationship between UBN CRU precipitation and the ISM index is positive, indicating that a stronger Indian Monsoon is associated with more precipitation in the UBN. This result is consistent with the long recognized link between the Indian Monsoon and East Africa (Walker, 1910; Walker and Bliss, 1932; Camberlin, 1997), which is generally attributed to the fact that a strong Indian Monsoon results in low pressure in the equatorial Indian Ocean, which in turn influences the advection of moisture into Ethiopia. The relatively weak statistical association between ISM and UBN precipitation in our analysis might be the result of averaging over the entire season (JJAS) and/or the choice of time period and indices.

In EQL, teleconnections are generally more difficult to characterize, particularly for the MAM long rains. Our results show no statistically significant relationship between CRU precipitation and the four large scale indices considered in MAM, which is not surprising in light of previous studies of these rains (Camberlin *et al.*, 2009). In OND, we do see a highly significant association between EQL rains and evolutionary phases of IOD, with a positive phase IOD leading to more precipitation in EQL. This relationship with the Indian Ocean is consistent with previous studies (Goddard and Graham, 1999; Black, 2005; Funk *et al.*, 2014). Proper representation of this association might be particularly important for climate change scenarios in EQL and the GHA more generally, as trends in Indian Ocean SST patterns and atmospheric convergence have been identified as likely drivers of 21st century precipitation change in the short rains (Cook and Vizy, 2013; Funk *et al.*, 2014). Our CRU results also show some evidence of a positive correlation between ENSO and EQL OND precipitation, which is consistent with previous studies and has been attributed in part to the ENSO influence on the IOD (Black, 2005). The ENSO relationship with OND precipitation is not statistically significant in our analysis.

Evaluation of teleconnections in the CMIP5 models indicates that a model's ability to replicate statistics of precipitation (Section 3.1) is often unrelated to the model's representation of known teleconnections. In the UBN, for example, CSIRO and GFDL offered the best match to mean CRU precipitation (Table 1), but CSIRO differs significantly from observation on the strength of ENSO and ISM influence on the region while GFDL shows weak association with ENSO and GSST and no association with ISM. CCSM4, which provided the best combination of mean precipitation and precipitation variability statistics, differs from observation in the sign and significance of association with ISM and IOD and shows relatively weak ENSO association, though it does match observed GSST influence. In contrast, HadGEM, IPSL, and (to some extent) MIROC5 all capture the sign and approximate strength of ENSO and GSST influence. MIROC5 also agrees with observed results for ISM and IOD, while HadGEM and IPSL show less consistency for these weaker drivers. Other models, including BCC and GISS, show the wrong sign of ENSO influence, while BCC and CanESM show the wrong sign of GSST influence. This suggests that precipitation from these models (even when bias corrected and scaled) should not be used in future climate projections, as changes in GSST and ENSO are two of the most important potential drivers of precipitation change in coming decades. We emphasize that all GCM teleconnection calculations were made using indices and precipitation extracted from the same model simulation.

For EQL, the top performing models in terms of statistics of precipitation (BCC and CESM for MAM precipitation, GISS for OND precipitation, and CSIRO for seasonality) do not always excel in the representation of teleconnections. BCC shows statistically significant differences from CRU on the ENSO and IOD influence in MAM while GISS does not capture the strength of IOD influence in OND. CSIRO differs significantly from observation on the influences of GSST, ISM, and IOD in OND. CESM performs reasonably well for MAM teleconnections, in that there are no statistically significant differences between CESM and observed correlations, but several other models – most notably HadGEM and CCSM4 – provide a better match to observed teleconnections across both MAM and OND. Given the complex nature of teleconnections in EQL, especially in MAM, it is not surprising that a number of models are at odds with observations. CanESM, GFDL, and IPSL, for example, have a tendency to overestimate the influence that large‐scale drivers have on variability in MAM precipitation. This suggests that MAM precipitation in these models is too tightly coupled to variability in the Indian and Pacific Oceans, but a full explanation would require analysis of model dynamics at sub‐seasonal scale.

### 3.3 Continental scale variability

**Figure 4:**
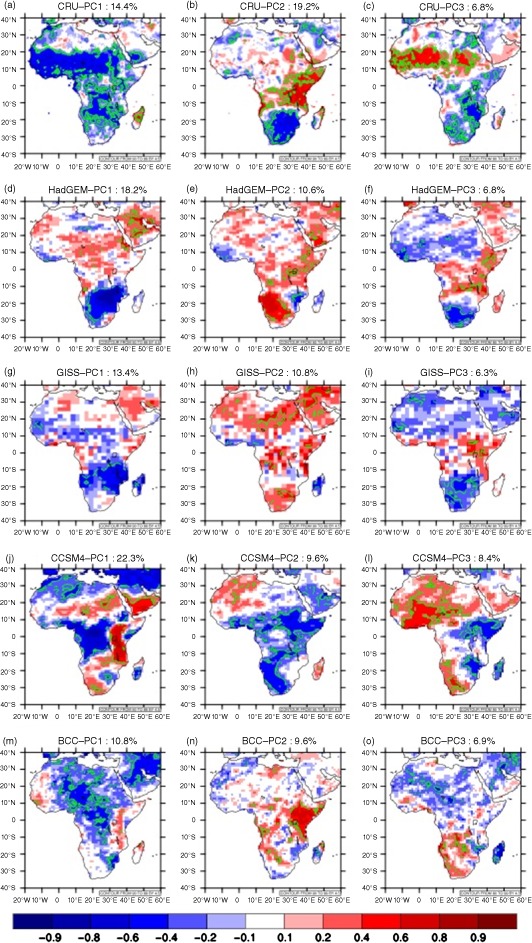
Spatial displays of the three leading principle components of an analysis performed on annual mean (July‐June) precipitation over Africa for the period 1950–1995: (a–c) CRU, (d–f) HadGEM, (g–i) GISS; (j–l) CCSM4, (m–o) BCC. Shading indicates correlation between the PC and precipitation at each grid cell, and contours (green in online) indicate 95% significance, accounting for temporal autocorrelation. Percentage of variability explained by each PC is shown in the figure.

**Figure 5:**
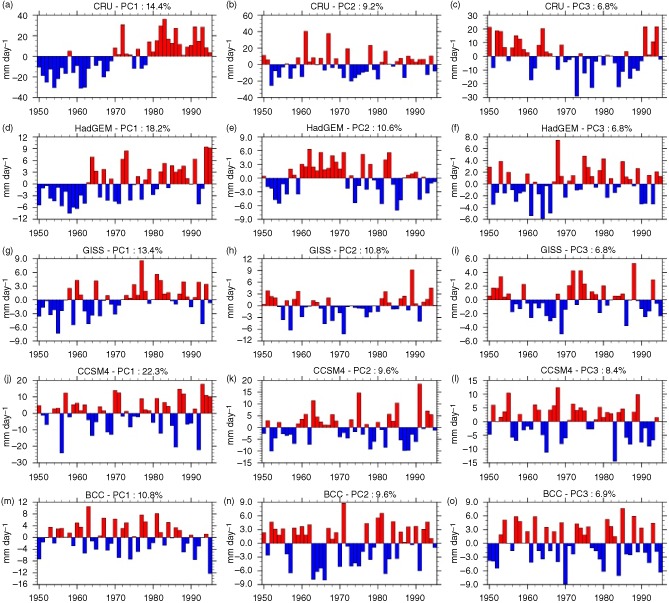
Time series of PCs mapped in Figure 4. (a–c) CRU, (d–f) HadGEM, (g–i) GISS; (j–l) CCSM4, (m–o) BCC. Percentage of variability explained by each PC is shown in the figure.

**Figure 6:**
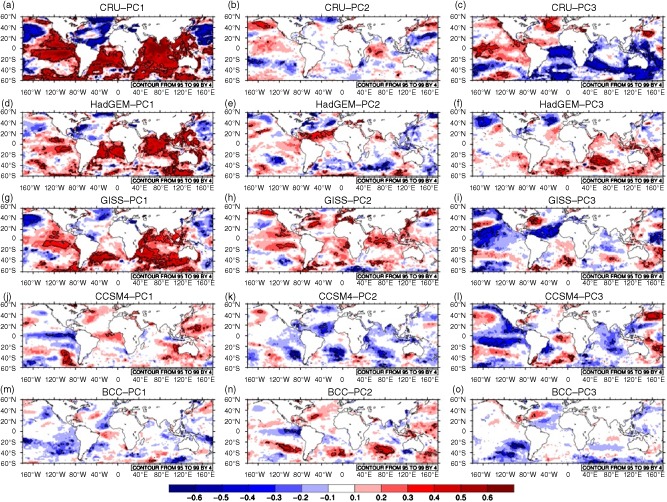
Linear correlation between annual sea surface temperature of HadISST, 60°S‐60°N, and the PC time series shown in Figure 5 for the period 1950–1995: (a–c) CRU, (d–f) HadGEM, (g–i) GISS; (j–l) CCSM4, (m–o) BCC.

Yet another approach to evaluating CMIP5 performance is to consider how each model represents major patterns of variability at continental scale. For example, as shown in Figure 4(a)–(c) (and shown in a similar analysis in Giannini *et al.* (2008)), the first three principal components of variability for African precipitation at annual timescale represent (1) continent‐wide wetting and drying, (2) opposing variability in southern Africa and the Gulf of Guinea Coast *versus* the rest of the continent, and (3) opposing variability in the Sahel *versus* the rest of the continent. The first mode of variability exhibits a positive trend (Figure 5(a)) that can be associated with pan‐tropical increases in SST (Figure 6(a)). Modes two and three, meanwhile, have been associated with ENSO activity (Giannini *et al.*, 2008). We note that in our analysis PC2 correlates with both western Pacific (El Nino) warming and a gradient in Indian Ocean SST (Figure 6(b)) while PC3 correlates with an El Nino like signal in the Pacific and a tropical Atlantic SST anomaly suggestive of variability in the Atlantic Meridional Overturning (AMO) circulation. Indeed, temporal variability in PC3 is multi‐decadal (Figure 5(c)), which is consistent with AMO timescales of variability as well as with observed periods of drought and wet conditions in the Sahel.

None of the GCMs included in this study replicate the spatial structure of these PCs particularly well (Figure 4(d)–(o); for conciseness we show only four selected models). PC analysis is not a perfect diagnostic tool: the method enforces orthogonality, which complicates physically based explanation, and in this application it is sensitive to the fact that the analysis period of 1950–1995 might not capture a full picture of long‐term variability in the CMIP5 models. Nevertheless, certain aspects of the continental‐scale PC analysis are quite relevant to GCM evaluation. For example, observations indicate that a PC associated with widespread oceanic warming and continent‐wide drying explains nearly 15% of observed variance in annual precipitation. This could be an anthropogenic warming signal or a multi‐decadal oscillation. In either event, it would be reassuring if GCMs were able to capture this tendency.

While the spatial structure of precipitation correlations with leading PCs is not a perfect match in any of the GCMs included in this analysis, certain models do have lead PCs that resemble the temperature signature and SST correlations seen in observations. HadGEM and GISS stand out as examples of models in which the first PC exhibits a trend similar to observations and correlates within‐phase SST variability across the tropical oceans, albeit with greater interannual variability than in CRU observations (Figures 5(d) and (g) and 6(d) and (g)). The spatial association with precipitation (Figure 4(d) and (g)) is not as strong or coherent as in CRU, but the direction of correlation is correct across much of the continent in GISS and in the southern half of the continent for HadGEM. There is also evidence in HadGEM PC2 and GISS PC2 and PC3 of decadal scale variability resembling that seen in CRU PC3, which is similarly associated with tropical Atlantic SST gradients and patterns of warming and cooling in the western Pacific and Indian Ocean. This suggests that these models are capable of capturing the character of continental scale precipitation variability, even if the associations are not represented correctly in all regions. Other models capture some of these features, but to a lesser degree. In CanESM and MIROC5, for example, it is difficult to identify patterns of long‐term variability in the leading PCs and their associated correlations with precipitation and SST (Figures 4(j)–(o), 5(j)–(o), and 6(j)–(o)). CCSM4 has a coherent drying signal in PC2, but it is not associated with any temporal trend (Figure 5(k)) and is, in fact, correlated with cooler tropical SSTs (Figure 6(k)) rather than warmer.

While this analysis is qualitative, it does provide a view on which CMIP5 models most closely resembles observations across the entire continent. The relatively strong performance of HadGEM and GISS suggest that these models could be good candidates for downscaling. For dynamical downscaling, in which a regional climate model is nested inside the GCM, the fact that HadGEM and GISS capture the dominant relationships between SST and continental‐scale precipitation is an indicator that large‐scale atmospheric processes in the model might connect Africa to appropriate centres of climate action. A properly implemented regional climate model could ingest this information as boundary condition and potentially correct regional errors in the GCM field through higher resolution and regionally optimized physical parameterizations. For statistical downscaling, one must distinguish between methods that employ in‐region predictors for the downscaling process – for example, Bias Correction and Statistical Disaggregation (BCSD) or the Statistical DownScaling Model (SDSM) – and methods that generate predictions on the basis of large scale fields that can be remote from the area of interest – for example, empirical–statistical downscaling (ESD) (Benestad *et al.*, 2008; Winkler *et al.*, 2011). The former places greater demand on the GCM, since it requires that the GCM properly represents the connection between large scale centres of climate action and the region of interest – e.g. the GCM must both capture ENSO and represent its influence on circulations affecting East Africa. For the latter, it is possible to generate meaningful downscaled climate fields from any GCM that captures large scale variability in centres of climate action, even if the GCM fails to represent the dynamics of the teleconnection to East Africa. The relative strength of continental‐scale patterns in HadGEM and GISS is also encouraging, though not required for ESD if local precipitation is not included as a predictor.

### 3.4 Implications for climate projection

Table 3 lists the top performing models according to metrics of mean precipitation, interannual precipitation variability, precipitation seasonality, precipitation teleconnections, and continental scale patterns of precipitation variability. For regional mean, variability, and seasonality we simply list the top three performing models according to their agreement with CRU. This should not be read as a definitive or formal ranking, as we have used only a single metric for one time period compared against a single observational dataset. The purpose is simply to show the diversity of top performing simulations and to consider implications for how model selection would influence precipitation projections. For teleconnections, we define the top performing simulations as those that have the closest agreement with CRU correlations when averaged across the four large scale drivers considered in this study. Again, we do not suggest that this is the only or the best way to rank models based on teleconnections; it is simply an example. For continental scale patterns we list only HadGEM and GISS, as they were the only two models of the ten that we included in our comparison that provide a reasonable approximation of the observed continental‐scale variability patterns.

**Table 3:** Projected percent change in precipitation for the period 2040–2059 and 2080–2099, both relative to the 1950–1995 baseline, for RCP8.5

Selection criterion	Region/season								
	EQL:MAM			EQL:OND			UBN:JJAS		
	Models	2040–2059 (%)	2080–2099 (%)	Models	2040–2059 (%)	2080–2099 (%)	Models	2040–2059 (%)	2080–2099 (%)
Mean	CESM, BCC, CanESM	14.1	**28.7**	GISS, IPSL, HadGEM	**−20.9**	−18.6	GFDL, CSIRO, HadGEM	**−2.2**	−2.7
Variability	HadGEM, MIROC5, GISS	**−6.1**	−3.2	GISS, BCC, MIROC5	−4.6	−0.4	MIROC5, IPSL, CCSM4	−8.4	2.2
Seasonality	CSIRO, MIROC5, CanESM	9.0	**18.0**	CSIRO, MIROC5, CanESM	6.8	**16.0**	MIROC5, IPSL, HadGEM	−10.3	−0.1
Teleconnection	HadGEM, CCSM4, MIROC5	2.6	**9.5**	CCSM4, HadGEM, CanESM	9.4	**16.4**	MIROC5, IPSL, HadGEM	−10.3	−0.1
Continental patterns	HadGEM, GISS	**−7.6**	−5.0	HadGEM, GISS	**−10.2**	−7.3	HadGEM, GISS	**−5.2**	−1.5
None	All	0.2	9.6	All	−1.9	4.6	All	1.4	8.1

Values are shown for the average of the top three performing models according to each potential selection criterion considered in this paper, as evaluated on the basis of similarity to observation in CRU and supporting observational datasets. In each category the top three models are selected except for continental patterns, for which only two models exhibited particularly realistic behaviour. Values in bold indicate agreement in the sign of change in all selected models. All model data are linearly interpolated to a common 1° × 1° grid box.

For each combination of high performing models, we calculate the average predicted percent change in precipitation for EQL rainy seasons and the UBN rainy season under a high emissions scenario (RCP8.5). For comparison, we also calculate the percent change according to a flat average of all ten GCM simulations included in the study. This ten model average projects that changes in precipitation will be small through the mid‐21st century, and that in the second half of the 21st century there will be an increase in precipitation in both Nile headwaters regions. This is roughly consistent with multi‐model ensemble average results presented in the 5th Assessment Report of the IPCC, and is the result of averaging across models with widely divergent projections in the positive and negative direction. There is a statistical advantage in this kind of averaging, as it minimizes the influence of outliers without arbitrarily removing them from consideration. Nevertheless, it is interesting to consider how different selection criteria might influence the average projection for the Nile River basin.

As is evident in Table 3, different selection criteria yield different sets of top performing simulations, and even within a single selection criterion there is no strong tendency towards model consensus on the direction of projected precipitation change. In EQL MAM, it is interesting to note that the models that best replicate observed mean and seasonality in precipitation – i.e. the models that would be judged to be most realistic in precipitation climatology – tend to project larger positive changes in precipitation than the full model average. Models that capture observed teleconnections most realistically (according to our simplified metric of evaluation), in contrast, agree relatively closely with the all‐model average. This provides some measure of confidence in the multi‐model ensemble projection for this region, since it is consistent with what is predicted by models that replicate observed influences of large‐scale forcing on precipitation (noting, however, that these influences are weak in the observational analysis). Models selected on the basis of continental patterns of variability project a decrease in precipitation, particularly for the mid‐21st century, but this selection criterion is intended to identify models with value for downscaling analysis rather than models that capture any particular aspect of local precipitation correctly.

For EQL OND the divergence of model projections is even more evident. Simply selecting model realizations based on their representation of mean precipitation in the OND rainy season would lead to a projection of significant *decreases* in precipitation, in contrast to the all‐model average. Selecting models that capture EQL seasonality correctly or that match observed teleconnections would lead to the opposite conclusion.

In the UBN, meanwhile, it is interesting that every selection criterion yields an average projection for small to moderate decreases in precipitation for 2040–2059 and for very little change from baseline in 2080–2099. This is in contrast to the all‐model average projection of late‐21st century increases in precipitation. Read literally, the implication of this result is that the high end of the all‐model precipitation projection is populated by GCM realizations that do not excel in any metric of evaluation considered in this paper. This reduces our confidence in the all‐model projection and suggests that the physical basis for projected precipitation change in each GCM needs to be examined further. At the same time, we note that MIROC5 and IPSL, two models that perform relatively well according to multiple evaluation metrics, disagree on the direction of 21st century precipitation change in this region. So there is significant uncertainty even between models selected for strong historical performance in the UBN.

The clearest implication of these results is that the ensemble mean projection should be interpreted with great caution. Not only is there a large spread between models, but the ensemble mean may be strongly influenced by model realizations that do not provide particularly good performance against any metric used in this paper. Users of GCM output who communicate directly with decision makers would be advised to adopt a Robust Decision Making framework (Lempert *et al.*, 2006), in which a full range of potential outcomes are presented, rather than a projection based on the ensemble mean. For more sophisticated GCM users, the differences between evaluation metrics can provide some guidance about how to downscale CMIP5 GCM realizations. A model that exhibits a strong precipitation bias, for example, might still provide reasonable representation of large scale teleconnections and could therefore be useful in dynamical downscaling or ESD, while a model that captures the mean but does so while missing all major teleconnections might be a less promising candidate for downscaling.

## 4 Conclusions

A number of individual findings presented in this paper are consistent with previously published analyses. Similar to Jury (2015) and Otieno and Anyah (2013), we find that CMIP5 models can be ranked on the basis of precipitation statistics, but that in East Africa there is no strong consistency in model performance across metrics. Further, our results reinforce findings for Rowell (2013) that CMIP5 models differ strongly in their representation of teleconnections to Africa at regional scale, and we find that a ranking based on representation of these teleconnections would be quite different from a ranking based on precipitation statistics. At continental scale, we find patterns of variability similar to Giannini *et al.* (2008) in observations, and here we extend that analysis to examine precipitation variability and associated SST patterns in selected CMIP5 models. Taken together, these analyses highlight complexities and opportunities for evaluating GCM suitability for studies of climate change in the Nile River basin.

The goal of this study is not to provide comprehensive GCM evaluation or to offer a specific list of models that are ‘recommended’ for use in the Nile basin. Rather, our analysis is intended to examine aspects of GCM simulations at three distinct scales and conceptual frameworks: local precipitation statistics, teleconnections to a region of interest, and dominant modes of variability across a continent.

The application of the local statistics framework for model application and evaluation is quite common but applies to only a limited range of studies: those that are concerned with raw GCM precipitation fields or those that apply a simple bias correction and disaggregation methodology to correct GCM precipitation for impacts analysis. While this approach is not uncommon, and direct plots of GCM precipitation projections are still featured in influential climate reports such as the IPCC 5th Assessment Report (IPCC, 2013), there are many applications in which precipitation projections are made without any direct use of local GCM precipitation estimates. For example, local precipitation projections can be generated through dynamical and statistical downscaling that uses large‐scale GCM fields to derive a local projection (Benestad *et al.*, 2008; Winkler *et al.*, 2011).

The teleconnections analysis presented in this paper leans towards this framework, as it considers a GCM's ability to capture relationships between regional scale precipitation and large‐scale forcings. The analysis does still depend on GCM precipitation in the region of interest, but only on relative variability in the precipitation field and not on absolute value. A GCM that captures such teleconnections, then, might be of value in dynamical downscaling or in statistical downscaling that uses within‐region predictor fields (e.g. Wilby *et al.*, 2002), because the model is able to connect large scale drivers to local variability.

Finally, the qualitative continental analysis presented here offers a view on which GCMs offer meaningful representation of African precipitation variability at large scale. A model that performs well by this standard could be useful for dynamical downscaling studies that use a relatively large RCM domain or for statistical downscaling methods that are based only on remote teleconnections: even if the GCM fails to represent precipitation variability correctly within the specific region of interest, it can capture the global to continental scale processes that influence RCM boundary conditions or the predictors in a teleconnections‐based projection. As noted earlier, all of these results have to be interpreted as the combined product of differences between GCMs and differences between specific model realizations due to long‐term internal variability in the climate system. We have used only a single ensemble member for GCM, and the small number of realizations included in the CMIP5 archive would limit our ability to characterize statistics of internal variability even if we used all available ensemble members. Ongoing studies that use large ensembles of single GCMs will provide further insights on this problem (Kay *et al.*, 2014).

For the Nile Basin specifically, our analyses warn against using simple precipitation statistics to select GCMs for impacts analysis, as many of the models that rank best according to those metrics fail to capture observed teleconnections with GSST and other large‐scale climate modes that might evolve under global warming. The continental scale analysis reinforces this conclusion, as models that capture the dominant modes of observed African precipitation variability (GISS and HadGEM) have relatively poor precipitation statistics and teleconnections for some aspects of Nile headwaters precipitation. Nevertheless, for certain kinds of downscaling analyses these models might be more useful for climate projection than models with more attractive historical precipitation statistics.

At the same time, this study does suggest that informed selection of GCMs can be valuable for regional climate studies of the Nile, provided that the user is clear on the objectives of the study and relevant metrics. In the UBN, for example, it is notable that the all‐model average projection of precipitation change is larger than the average of models selected according to any of the criteria considered in this paper. The multi‐model mean projection, then, might overestimate future precipitation in this critical Nile headwaters region. For this reason the evaluation, selection, and application of CMIP5 projections for use in the Nile – and for any region with wide spread in the multi‐model GCM ensemble – requires careful consideration, and should be based on metrics specifically relevant to the goals of analysis.
